# Cigarette smoking as a risk factor for auditory problems

**DOI:** 10.1016/S1808-8694(15)30556-5

**Published:** 2015-10-19

**Authors:** Carolina Pamplona Paschoal, Marisa Frasson de Azevedo

**Affiliations:** 1 MSc in Sciences, Federal University of São Paulo, Speech and Hearing Therapist; 2 PhD in Human Communication Disorders, Federal University of São Paulo, Adjunct Professor and Vice-Head of the Hearing Disorder Program, Federal University of São Paulo. Federal University of São Paulo

**Keywords:** audiometry, high-frequency, hearing loss, smoking, otoacoustic emissions

## Abstract

Smoking is a public health concern and we are still unsure of its relation with auditory problems.

**Aim:**

To study the effects of cigarette smoking in auditory thresholds, in otoacoustic emissions and in their inhibition by the efferent olivocochlear medial system.

**Materials and Methods:**

144 adults from both genders, between 20 and 31 years of age, smoking and non-smoking individuals were submitted to conventional and high-frequency audiometry, transient stimuli otoacoustic emissions and suppression effect investigation.

**Results:**

smokers presented worse auditory thresholds in the frequencies of 12.500Hz in the right ear and 14,000 kHz in both ears. Regarding the otoacoustic emissions, smokers group presented a lower response level in the frequencies of 1,000Hz in both ears and 4,000Hz in the left ear. Among smokers there were more cases of cochlear dysfunction and tinnitus.

**Conclusion:**

Our results suggest that cigarette smoking has an adverse effect on the auditory system.

## INTRODUCTION

Smoking is a serious public health problem. Several studies have shown its many harmful effects. Smokers, when compared to non-smokers, are at a greater risk of having bacterial respiratory infections and both acute and chronic viral diseases; oral, laryngeal, esophageal, pancreatic, renal, and bladder cancer; circulatory diseases such as arteriosclerosis, aortic aneurism, stroke, and multiple organ disorders^1^.

Tobacco toxicity is directly related to the number of cigarettes smoked and inversely related to the age at which the habit was initiated^2^.

Various studies in the literature have shown that smoking is considered to be a risk factor for the development of conductive^3^,^4^ and sensorineural^5^, ^6^, ^7^, ^8^ hearing losses, with widely diverse outcomes.

In California, increased auditory thresholds at 4000Hz were found among smokers^5^, while in Malaysia such thresholds were found at 6000Hz^6^. Another study concluded that smoking workers exposed to noise are more predisposed to acquiring hearing losses at 3000 and 4000Hz^7^.

A longitudinal Trial reported that the risk of acquiring hearing loss, mainly at high frequencies, is directly related to the number of cigarettes a day a smoker has and the time for which the subject has been a smoker^6^,^8^. Former smokers tend to develop high frequency hearing loss, thus suggesting that the harmful effects of smoking upon hearing are cumulative and permanent^8^.

A study carried out with animals in a laboratory observed the infliction of cochlear damage after exposure to cigarette smoke^9^. Another study using animals found nicotine receptors in hair cells, thus indicating that smoking may have direct ototoxic impact upon hair cell function and reduce the potential of the hearing neurotransmission organ ^10^. As less oxygen is available to the organ of Corti, there is less energy available for the cochlea and possibly more hair cell injury^9^,^11^,^12^.

Some smokers complain of tinnitus, an indication of peripheral or central disorders. Tinnitus has been associated with smoking, but only indirectly as it is a manifestation seen in smokers with hearing loss^11^.

According to the literature, smoking is associated with lower blood oxygen levels, vascular obstruction, altered blood viscosity, and possibly ototoxicity. But it is not known how much of it impacts the auditory system. There is controversy as to whether cigarettes can really be deemed as a risk factor for the development of hearing loss.

There are several tests to assess the peripheral auditory system. Conventional tone threshold audiometry allows the investigation of pure tones at 250 and 8000Hz. High frequency tone threshold audiometry looks into pure tones above 8000Hz and assists in the assessment of basal cochlear response. One of the main clinical applications of high frequency audiometry is hearing monitoring, as it is a valuable tool in assessing early auditory involvement^13^.

Otoacoustic emission studies provide direct insight into outer hair cell cochlear amplification; OAEs indicate middle ear integrity and normal cochlear biologic mechanism activity. The efferent innervation is made up by a large number of fibers that form the medial and lateral efferent system. The medial efferent system is connected to the innervation of outer hair cells, while the lateral system is related to inner hair cells. The release of acetylcholine in the synaptic cleft through the medial olivocochlear efferent tract modulates the motion of outer hair cells 14, 15, 16.

Efferent olivocochlear system function can be assessed by comparing the response levels of transient otoacoustic emissions with and without contralateral auditory stimulation. Normal efferent auditory pathway function presents reduced OAE response when contralateral auditory stimulation is present. Such effect is brought about by the medial olivocochlear tract, as it attenuates cochlear amplification gains and reduces cochlear membrane motion through outer hair cell synapses. This method enables the assessment of the impact of efferent neural activity on the cochlea^17^.

Therefore, this study aims to verify the assumption that smoking has harmful effects upon hearing and alters audiometric thresholds in the occurrence of transient otoacoustic emissions and in the inhibition of them by the medial efferent olivocochlear system.

## MATERIALS AND METHOD

This study was carried out in the audiology ward and approved by the Research Ethics Committee at our institution under permit CEP0447/05. College students meeting the criteria were invited to participate. All subjects involved in the study have read the informed consent term on the conduction of the study and information disclosure, according to ordinance 196/96.

This study looked into auditory thresholds (hearing level at 250 to 16000Hz), outer hair cell function, and medial efferent olivocochlear systems in 144 adults of both genders, with ages ranging between 20 and 31 years, 72 smokers and 72 non-smokers, paired by gender and age.

For this study, smokers are individuals who smoke five or more cigarettes a day for one year or more, as proposed by Cruickshanks et al. (1998)^18^.

Former smokers, individuals with middle ear disorders, subjects complaining of metabolic disorders, hormonal disorders, noise-induced hearing loss, drug-induced hearing loss were excluded after being interviewed.

Evaluation Procedure:

1. Interview performed directly with the patient through simple questions and “Yes or No” answers, and further clarification when needed.
2. Acoustic impedance measures to verify middle ear integrity: tests used the Interacoustics AZ-7 device. Curves were categorized according to JERGER (1970) into A, Ad, Ar, B, C. Individuals with “A” tympanometric curves were enrolled in the study.
3. Conventional (250 to 8000Hz) and high frequency (10000 to 16000Hz) audiometry: tests were performed in a soundproof booth using a Grason Stadler GSI 61 audiometer in accordance with the following standards: ANSI S3.6-1989; ANSI S3.43-1992; IEC 645-1 (1992); IEC 645-2 (1993); UL 544. Conventional audiometry required the use of Telephonics TDH-50P earphones with 80 ohms of impedance; high frequency audiometry required the use of Sennheiser HAD-200 earphones with 40 ohms of impedance.
4. Transient otoacoustic emissions (TOAEs): tests were performed in a silent environment, with individuals kept quiet in a soundproof booth and well-fitted with a probe. Software program Fullmenu (20ms) from ILO292 Otodynamic Analyser, release 4.2 was used. Responses were considered present when they were 3 dB above noise at 1000, 2000, 3000 and 4000 Hz (Dolhen et al., 1991). Suppression effect was analyzed through linear click stimulation at 65 dBpeNPS and with the introduction of white noise at 60 dB NPS in the contralateral ear, using a TDH-39 earphone from audiometer MAICO MA-17, standard ANSI-69. Suppression was deemed present when reductions were greater than or equal to 0.5 dB in the TOAE response amplitude with contralateral noise when compared to TOAEs without contralateral noise (Collet et al.,1990).

The data sets gathered for the smoker group (S) were compared to those of non-smokers (NS). In the analysis of the smoker group (S) the number of cigarettes smoked per day and the time for which the subject has smoked were taken into account. Another study^18^ used the terminology “packs-years,” corresponding to the number of cigarettes per day divided by 20 (number of cigarettes per pack), multiplied by the number of years for which the subject has smoked. This is how the time of smoking was quantified.

Non-parametric tests were used in statistical analysis to find whether statistically significant differences were present between smokers and non-smokers in relation to the following items: occurrence of tinnitus; hearing level at 250 to 8000 Hz (conventional audiometry); hearing level at 10000; 12500; 14000 and 16000Hz (high frequency audiometry); TOAE response; presence of suppression. The following statistical tests were used: Student's t, Mann-Whitney, and the two ratio equality test.

In order to verify the existence of an association between the high frequency audiometry findings and the time for which the subject has smoked, a study was done correlating “pack-years” and high frequency audiometry test results. The tests used for statistical purposes were Spearman's ratio and the correlation ratio test. PThe following categorization scale was used in the Spearman test: 0% to 20% - bad; 20% to 40% - poor; 40% to 60% - regular; 60% to 80% - good; 80% to 100% - great.

A significance level of 5% (0.05) was adopted in this study.

## RESULTS

This study looked into 144 adult patients of both genders aged between 20 and 31 years, 72 smokers (S) and 72 non-smokers (NS) paired for gender and age.

Regardless of the outcomes of the procedures performed during the study (conventional and high frequency audiometry, TOAE with and without contralateral noise), one piece of data stood out in the interview): tinnitus prevalence a rates.

Tinnitus was found in 29 (40.3%) individuals in the smoker group (S) and in 8 (11.1%) individuals in the non-smoker (NS) group (p<0.001 according to Student's t-test).

Mean thresholds observed in conventional audiometry ranged between 2.01 and 6.39 dBNA; median values ranged between 0 and 5; and standard deviations between 2.61 and 4.72. Smoker threshold values were worse than non-smokers in all frequency ranges. The Mann-Whitney test failed to find statistical differences except for left ears at 8000Hz with p=0.016 (S group: mean 4.51; median 5; standard deviation 3.48 - NS group: mean 3.26; median 2.5; standard deviation 3.86).

The possible correlation between conventional audiometry thresholds and tinnitus was investigated. The Mann-Whitney test was used to compare subjects with and without tinnitus. Subjects with tinnitus had worse threshold values in their right ears. The p values for frequencies of 250, 500, 1000, 4000, 6000 and 8000Hz were lower than 0.001 (p<0.001); whereas for 2000Hz p=0.011 and 3000Hz p=0.008. As far as left ears are concerned, subjects with tinnitus also had worse threshold values and all frequencies had p<0.001.

Mean threshold values observed in high frequency audiometry (mean and median) and their standard deviations for smokers and non-smokers are presented in Table 1. The results of statistical analysis (Mann-Whitney) are to the right of the Table.

**Table 1:** Auditory thresholds (dBNA) in high frequency audiometry in both ears comparing smokers (S) and non-smokers (NS).

	Frequency (Hz)		Mean (dBNA)	Median (dBNA)	Standard Deviation	N	p-value(Mann-Whitney)
RE	10000Hz	Group S	19,17	20	7,22	72	0,320
		Group NS	17,43	17,5	4,60	72	
	12500Hz	Group S	25,49	25	6,77	72	<0,001*
		Group NS	20,83	20	3,56	72	
	14000Hz	Group S	31,32	30	7,12	72	0,004*
		Group NS	27,78	30	4,19	72	
	16000Hz	Group S	37,57	35	9,49	72	0,111
		Group NS	33,89	35	4,76	72	
LE	10000Hz	Group S	19,10	15	7,19	72	0,094
		Group NS	16,67	15	4,11	72	
	12500Hz	Group S	22,85	20	7,54	72	0,221
		Group NS	20,69	20	4,77	72	
	14000Hz	Group S	29,65	30	7,52	72	0,081
		Group NS	27,15	25	4,95	72	
	16000Hz	Group S	35,90	35	9,21	72	0,259
		Group NS	32,85	35	4,51	72	

Legend: Group S = group of smokers

Group NS = group of non-smokers

N = number of individuals

* p<0.05 (significant)

The correlation between high frequency audiometry thresholds and occurrence of tinnitus was also investigated. Test results for subjects with and without tinnitus were compared using the Mann-Whitney test. On right ears, subjects with tinnitus had worse thresholds. The p values for the 10000, 12500 e 16000Hz frequencies were lower than 0.001; at 14000Hz p=0.002. On left ears, subjects with tinnitus also had worse thresholds, and the p values for all frequencies were under 0.001.

Transient otoacoustic emissions (TOAEs) were statistically analyzed for both groups (smokers and non-smokers) taking occurrence (Table 2 - right ear and Table 3 - left ear) and overall response values (OR) into account by TOAE frequency range (1000 to 4000Hz) (Table 4 - right ear and Table 5 - left ear) using Mann-Whitney.

**Table 2:** Occurrence of transient otoacoustic emissions on right ears, overall response (OR) per frequency band from 1000 to 4000Hz, comparing smokers (S) to non-smokers (NS).

	TOAE		Group S		Group NS		p-value (Mann-Whitney)
RE			N	%	N	%	
	OR	Present	64	88,9	71	98,6	0,016*
		Absent	8	11,1	1	1,4	
	1000Hz	Present	71	98,6	72	100	0,068
		Absent	1	1,4	0	0	
	2000Hz	Present	71	98,6	72	100	0,068
		Absent	1	1,4	0	0	
	3000Hz	Present	66	91,7	71	98,6	0,032*
		Absent	6	8,3	1	1,4	
	4000Hz	Present	64	88,9	71	98,6	0,016*
		Absent	7	11,1	1	1,4	

Legend: Group S = group of smokers

Group NS = group of non-smokers

N = number of individuals

* p<0.05 (significant)

**Table 3:** Occurrence of transient otoacoustic emissions on left ears, overall response (OR) per frequency band from 1000 to 4000Hz, comparing smokers (S) to non-smokers (NS).

	TOAE		Group S		Group NS		p-value (Mann-Whitney)
LE			N	%	N	%	
	OR	Present	64	88,9	71	98,6	0,016*
		Absent	8	11,1	1	1,4	
	1000Hz	Present	71	98,6	72	100	0,074
		Absent	1	1,4	0	0	
	2000Hz	Present	70	97,2	71	98,6	0,063
		Absent	2	2,8	1	1,4	
	3000Hz	Present	64	88,9	71	98,6	0,016*
		Absent	7	11,1	1	1,4	
	4000Hz	Present	64	88,9	71	98,6	0,016*
		Absent	7	11,1	1	1,4	

Legend: Group S = group of smokers

Group NS = group of non-smokers

N = number of individuals

* p<0.05 (significant)

**Table 4:** Level (dB NPS) of overall response (OR) by frequency band from 1000 to 4000Hz to transient otoacoustic emissions (TOAE) on right ears (RE) comparing smokers (S) and non-smokers (NS).

TOAE			Mean	Median	Standard Deviation	N	p-value (Mann-Whitney)
RE	1000Hz	Group S	5,28	5	2,10	64	0,002*
		Group NS	6,48	6	2,24	71	
	2000Hz	Group S	7,23	9	2,64	64	0,071
		Group NS	7,98	7	2,20	71	
	3000Hz	Group S	8,47	9	2,66	64	0,553
		Group NS	8,92	9	2,80	71	
	4000Hz	Group S	8,86	9	3,03	64	0,076
		Group NS	9,86	9	2,89	71	
	OR	Group S	8,47	8,5	2,10	64	0,631
		Group NS	8,55	8,2	2,51	71	

Legend: Group S = group of smokers

Group NS = group of non-smokers

N = number of individuals

* p<0.05 (significant)

**Table 5:** Level (dB NPS) of overall response (OR) by frequency band from 1000 to 4000Hz to transient otoacoustic emissions (TOAE) on left ears (LE) comparing smokers (S) and non-smokers (NS).

	TOAE		Mean	Median	Standard Deviation	N	p-value (Mann-Whitney)
LE	1000Hz	Group S	5,72	5	2,06	64	<0,001*
		Group NS	7,27	7	2,51	71	
	2000Hz	Group S	7,84	7	2,70	64	0,337
		Group NS	8,31	8	2,72	71	
	3000Hz	Group S	9,10	9	2,81	64	0,884
			Group NS	9,80	9	2,76	71
	4000Hz	Group S	9,27	9	2,63	64	0,032*
		Group NS	10,20	9	2,41	71	
	OR	Group S	9,17	8,9	2,50	64	0,855
		Group NS	9,24	9,2	2,04	71	

Legend: Group S = group of smokers

Group NS = group of non-smokers

N = number of individuals

* p<0.05 (significant)

The occurrence of individuals with auditory tone thresholds by 25 dB NA on conventional audiometry (250 to 8000Hz) and absence of TOAEs characterized as cochlear disorder in the group of smokers amounted to 13.9% (10 individuals) and to 2.8% (2 individuals) for the non-smoker group (p=0.016). The two ratio equality test was used for statistical analysis.

The correlation between incidence of tinnitus and transient otoacoustic emissions was studied. Student's t-test was used to analyze the results of individuals with and without tinnitus. The comparison was run for the two groups (smokers and non-smokers) separately. In the smoker group, 30.6% (19) of the 62 individuals with present TOAE complained of tinnitus and 69.4% (43) did not; all ten individuals with absent TOAE complained of tinnitus. In the non-smoker group, 8.6% (6) of the 70 individuals with present TOAEs complained of tinnitus and 91.4% (64) did not; two individuals had absent TOAEs and complaints of tinnitus. Statistical analysis found p=0.032 for smokers and p=0.003 for non-smokers.

TOAE with contralateral noise led to reduced response levels (suppression) in all cases in either of the groups. The mean differences between the overall response of OAEs with and without contralateral noise (suppression) in the smoker and non-smoker groups are presented in Table 6. Statistical analysis (Mann-Whitney) is presented in the bottom of the Table.

**Table 6:** Mean suppression values for right and left ears comparing between smokers (S) and non-smokers (NS).

Suppression	RE		LE	
	Group S	Group NS	Group S	Group NS
Mean	3,14	2,46	3,38	2,52
Median	3,2	2,5	3,2	2,5
Standard Deviation	1,00	0,75	0,91	0,73
Minimum Value	0,8	0,7	1,2	1,2
Maximum Value	5,5	4,2	5,2	4,4
N	64	71	64	71
p-value (Mann-Whitney)	<0,001*		<0,001*	

Legend: Group S = group of smokers

Group NS = group of non-smokers

N = number of individuals

* p<0.05 (significant)

The correlation between “pack-years” and audiometry test results was investigated in order to verify the association between high frequency audiometry results and the time for which the subject has smoked (Tabela 7). The tests used in this statistical analysis were Spearman's ratio and the correlation ratio test. Graph 1 shows these correlations for 10000 and 16000Hz for right and left ears respectively.

**Table 7:** Correlation between “packs-years” (quantity/time for which subject has smoked) and high frequency audiometry test results in both ears in smokers (S)

Packs-years			Spearman's ratio	Correlation ratio p-value
HF Audiometry	RE	10000Hz	69,4% R	<0,001*
		12500Hz	57,7% B	<0,001*
		14000Hz	52,1% B	<0,001*
		16000Hz	53,6% B	<0,001*
	LE	10000Hz	54,8% B	<0,001*
		12500Hz	66,6% R	<0,001*
		14000Hz	58,2% B	<0,001*
			56,6% B	<0,001*

Legend: R = regular corrlation (40-60%)

G = good correlation (60– 80%)

Group S = smokers

* p<0.05 (significant)

## DISCUSSION

The results presented in the study will be discussed in accordance with the scheme presented above.

The smoker group had significantly higher tinnitus prevalence rates. Tinnitus is a sound sensation produced in the absence of an external sound source. It is undoubtedly one of the main otologic manifestations found in the clinical practice^19^.

The literature provides a myriad of explanations to account for the mechanisms of tinnitus. Some authors reported that tinnitus is a frequent symptom in cochlear disorder, principally when the neuroepithelial structures of the organ of Corti are involved^20^.

A theory states that injuries to outer hair cells may lead to them being more triggered, thus stimulating nerve fibers the same way as if it were real sound and reducing the inhibition from the central nervous system (CNS) and increasing spontaneous neuronal activity in the auditory system^21^.

The idea that tinnitus is generated in a portion of the basilar membrane in which inner hair cells are preserved and outer hair cells are injured may explain the occurrence of tinnitus in individuals with normal hearing. Diffuse damage of up to 30% in outer hair cells may occur without detectable hearing loss^22^.

Audiological assessment of tinnitus patients should consist of audiometric threshold tests including frequencies above 8000Hz, impedance tests, and otoacoustic emissions in order to obtain a more accurate diagnosis^23^.

In this study, the tests mentioned above were carried out to try and clarify the effects smoking may have on the auditory system. However, with the increased prevalence of tinnitus identified among smokers, there came the doubt as to whether smoking can be considered as a direct cause of tinnitus or if tinnitus occurs due to changes in the auditory system introduced by the habit of smoking.

Some authors report an indirect association between smoking and tinnitus. Smoking is a risk factor for hearing loss mainly at high frequencies, and smokers had a higher incidence of tinnitus than non-smokers^11^.

In an attempt to better understand the relationship between smoking, tinnitus, and hearing disorders, tinnitus was analyzed based on the audiological evaluation data. In this case the groups were separated and auditory thresholds were compared for frequencies between 250 and 16000Hz to transient otoacoustic emissions in individuals with and without tinnitus.

We then found that both in the smoker and non-smoker groups the individuals with tinnitus had worse auditory thresholds from 250 to 8000Hz and greater incidence of absent emissions. The difference between individuals with and without tinnitus was statistically significant. Threshold values (250-8000Hz) between groups (smokers and non-smokers) were not significantly different. These findings suggest, as also reported in the literature11, that smoking is not directly associated with tinnitus and that tinnitus occurs due to the presence of hearing disorders. Tinnitus should thus be deemed as a manifestation (symptom) connected to disorders in the auditory system.

However, when looking at the associations with audiometric thresholds for frequencies ranging between 10000 and 16000Hz, test results have shown that individuals with tinnitus had worse auditory thresholds than individuals without tinnitus. This difference was statistically significant only among smokers. The smoker group also had worse thresholds for these frequencies in relation to the non-smoker group; this is possibly due to the impact smoking has in enhancing the onset of tinnitus.

When looking at the other test results (conventional and high frequency audiometry, TOAE, and TOAE suppression), on conventional audiometry (250-8000Hz), only at 8000Hz and on left ears was there a statistically significant difference in mean auditory thresholds, as smokers had greater values than non-smokers. On high frequency audiometry smokers had worse thresholds for all frequencies; statistically significant differences were found at 12500Hz on right ears and at 14000 Hz on both ears (Table 1).

According to cochlear physiology, each frequency in a sound wave stimulates a certain region of the cochlea. Stimulation is closer to the basal region of the cochlea the higher the frequency of the sound stimulation is. It is thus believed that conventional audiometry (250-8000Hz) assesses predominantly the medial portion of the cochlea, while high frequency looks into the basal portion of the cochlea^24^.

Differently from conventional tone threshold audiometry, no normality pattern has been established yet for audibility thresholds in high frequency audiometry. However, similarities were observed between the test results of non-smokers in our study and reports in the literature^13^,^25^.

Reports in the literature state that high frequency audiometry is very relevant in the early detection of sensorineural hearing loss and in monitoring the hearing capabilities of patients taking ototoxic medication, with kidney failure, presbycusis, and frequent exposure to noise. It is believed that initial alterations in high frequencies may precede hearing loss in conventional frequencies^13^,^23^.

There are few studies in the literature connecting smoking and hearing disorders. Studies report on a variety of results, probably due to differences in methodology. Apart from that, other factors should be taken into account in regards to studies done on smokers that are difficult to measure. The amount of nicotine and carbon monoxide taken in by smokers depends on a number of factors such as the characteristics of tobacco, rate of consumption and the habits of each individual (frequency, time and depth of smoke inhalation)^26^.

Noise exposure was not analyzed in this study, but it is worth mentioning that studies have shown that smokers exposed to noise presented higher probabilities of acquiring hearing losses (NIHL) than non-smokers exposed to the same noise levels^7^,^27^,^28^. Along the same line of thinking, some authors have reported that carbon monoxide (CO) enhances the effects introduced by noise levels that by themselves would not produce any alteration in auditory thresholds. In a study done with animals damage was observed more frequently on the base of the cochlea as test subjects were submitted to high frequency noise and carbon monoxide together with noise. More hair cells were damaged in the group exposed to both, with particular involvement of the inner hair cells. In exposure to carbon monoxide alone no changes were observed in inner or outer hair cells^29^.

A longitudinal study carried out with elderly patients, in an attempt to connect presbycusis and smoking, the authors reported involvement at 8000Hz^30^. Another similar study reported greater hearing involvement at 6000Hz in smoking elderly subjects^6^. A study done with younger populations reported that smoking harms hearing; involvement was observed at 4000Hz, and hearing losses were greater the higher was the smoker's tobacco intake8.

No studies were found in the researched literature in which high frequency audiometry (above 8000Hz) had been used as a means to assess smokers. However, in this study high frequency audiometry was used as it is a relevant tool to detect early hearing loss. Involvement in high frequencies alone, as found in our study, may suggest the onset of sensorineural hearing loss in smokers, who may in the future develop hearing loss in conventional frequencies. It is important to stress that the thresholds observed in smokers in high frequency audiometry showed a direct association with tobacco intake, as auditory thresholds got worse the higher was the quantity/time for which the subject smoked (Table 7, Graph 1). It is very likely that the effects of smoking appear first in the most basal portion of the cochlea, affecting high frequencies permanently and progressively.

In relation to transient otoacoustic emissions and suppression, the smoker group presented, on this study, statistically lower levels at 1000Hz in both ears and at 4000Hz in left ears and greater suppression of otoacoustic emissions (Table 2, Table 3, Table 4, Table 5, Table 6). Additionally, a statistically greater prevalence of cases of cochlear disorder (normal conventional audiometry results and absent otoacoustic emissions) was also found.

Otoacoustic emissions are a simple and fast method to assess the motility of outer hair cells. These cells are responsible for the motion of the basilar membrane through rapid and slow contractions, thus producing cochlear amplification^15^.

One of the methods used to investigate the function of the efferent olivocochlear system compares the level of response to transient otoacoustic emissions with and without contralateral auditory stimulation. Normal function efferent auditory pathways will present reduced response to otoacoustic emissions in the presence of contralateral auditory stimulation. The medial efferent olivocochlear system is responsible for such effect, as the synapses on the cochlear outer hair cells attenuate the cochlear amplification gain and reduce cochlear membrane motion. This method enables the assessment of the impact of efferent neural activity on the cochlea^17^.

A study looked at ten non-smoking subjects with normal bilateral hearing and submitted them to otoacoustic emission and brainstem auditory evoked potential tests after the administration of nicotine, only to realize that nicotine interferes with the neural transmission of auditory information. The authors reported that the effect of nicotine in higher neural centers may have an efferent inhibitory effect on outer hair cells due to an exacerbation on acetylcholine, the efferent auditory system neurotransmitter^31^. This could explain the increased suppression found among smokers.

Another study used mid-latency auditory evoked potentials as a means to assess the effects of smoking and withdrawal on the auditory system. A positive effect of smoking upon the central auditory system was observed as smokers maintained their smoking routine, suggesting an acceleration effect in neural processing. Conversely, withdrawal from smoking led to negative effects, indicating imbalances in the central auditory system^32^.

Reductions in the responses to otoacoustic emissions and increases in suppression indicate that smoking may have different effects on the peripheral and central auditory systems. More should be researched on the composition of tobacco and the possible effects of each substance may have to further clarify this matter.

The research literature offers some possible explanations to the effect tobacco has on the auditory system. Studies report that outer hair cells have nicotine receptors, suggesting that tobacco may have a direct ototoxic effect on outer hair cell function^9^,^10^. Another idea is that tobacco toxicity leads to reduced amounts of oxygen in the cochlear base, thus leading to degenerative injuries to the organ of Corti^8^. Studies observed animals exposed to carbon monoxide that developed outer and inner hair cell injury (more injury found in outer hair cells) and concluded that carbon monoxide causes concentrated damage on the more basal portion of the cochlea^30^. Another study reports that nicotine changes the blood characteristics, and such changes harm cochlear irrigation. The cochlear artery ends its irrigation at the site of the inner hair cells, and that is why tobacco introduces predominantly progressive hearing loss, being the higher frequencies involved first^12^.

In order to understand the effects of smoking on the auditory system, other studies need to be performed, principally those using evaluation procedures that may detect early hearing impairments such as high frequency audiometry, otoacoustic emissions, and auditory evoked potential. If possible, it would be interesting to measure the blood levels of tobacco constituting substances to relate each one of them to possible auditory disorders.

## CONCLUSION

This study indicates that smoking has harmful effects on hearing, as the smoker group had worse auditory thresholds in high frequencies (above 8000Hz), lower response levels to transient otoacoustic emissions, and higher suppression levels when compared to non-smokers. Additionally, the smoker group presented more individuals affected by tinnitus and cochlear disorders.
